# Immunogenicity and clinical effectiveness of the trivalent inactivated influenza vaccine in immunocompromised children undergoing treatment for cancer

**DOI:** 10.1002/cam4.596

**Published:** 2015-12-29

**Authors:** Rishi S. Kotecha, Ushma D. Wadia, Peter Jacoby, Anne L. Ryan, Christopher C. Blyth, Anthony D. Keil, Nicholas G. Gottardo, Catherine H. Cole, Ian G. Barr, Peter C. Richmond

**Affiliations:** ^1^ Department of Haematology and OncologyPrincess Margaret Hospital for ChildrenGPO Box D184PerthWestern Australia6840Australia; ^2^ Telethon Kids InstituteUniversity of Western AustraliaPO Box 855PerthWestern Australia6872Australia; ^3^ School of Paediatrics and Child HealthUniversity of Western AustraliaGPO Box D184PerthWestern Australia6840Australia; ^4^ Department of Infectious DiseasesPrincess Margaret Hospital for ChildrenGPO Box D184PerthWestern Australia6840Australia; ^5^ Department of MicrobiologyPathWest Laboratory Medicine WAPrincess Margaret Hospital for ChildrenGPO Box D184PerthWestern Australia6840Australia; ^6^ WHO Collaborating Centre for Reference and Research on InfluenzaVIDRLPeter Doherty Institute for Infection and Immunity792 Elizabeth StreetMelbourneVictoria3000Australia; ^7^ Department of PaediatricsPrincess Margaret Hospital for ChildrenGPO Box D184PerthWestern Australia6840Australia

**Keywords:** Cancer, chemotherapy, immunocompromised, influenza, pediatric, vaccination

## Abstract

Influenza is associated with significant morbidity and mortality in children receiving therapy for cancer, yet recommendation for, and uptake of the seasonal vaccine remains poor. One hundred children undergoing treatment for cancer were vaccinated with the trivalent inactivated influenza vaccine according to national guidelines in 2010 and 2011. Influenza‐specific hemagglutinin inhibition antibody titers were performed on blood samples taken prior to each vaccination and 4 weeks following the final vaccination. A nasopharyngeal aspirate for influenza was performed on all children who developed an influenza‐like illness. Following vaccination, seroprotection and seroconversion rates were 55 and 43% for H3N2, 61 and 43% for H1N1, and 41 and 33% for B strain, respectively. Overall, there was a significant geometric mean fold increase to H3N2 (GMFI 4.56, 95% CI 3.19–6.52, *P *<* *0.01) and H1N1 (GMFI 4.44, 95% CI 3.19–6.19, *P *<* *0.01) strains. Seroconversion was significantly more likely in children with solid compared with hematological malignancies and in children <10 years of age who received a two‐dose schedule compared to one. Influenza infection occurred in 2% of the vaccinated study population, compared with 6.8% in unvaccinated controls, providing an adjusted estimated vaccine effectiveness of 72% (95% CI −26–94%). There were no serious adverse events and a low reactogenicity rate of 3%. The trivalent inactivated influenza vaccine is safe, immunogenic, provides clinical protection and should be administered annually to immunosuppressed children receiving treatment for cancer. All children <10 years of age should receive a two‐dose schedule.

## Introduction

The last 40 years have seen marked improvements in the treatment for childhood cancer, with a significant reduction in mortality in developed countries 1, 2, 3, 4. For a number of childhood malignancies, increased cure rates have been achieved by increasing the intensity and duration of chemotherapy. However, this leads to prolonged periods of immunosuppression and vulnerability to infectious and toxic complications. In particular, influenza infection remains a significant cause of morbidity, mortality, and health expenditure among children undergoing treatment and within 6 months following the completion of therapy for cancer 5, 6, 7, 8, 9, 10.

Currently, annual vaccination with the inactivated influenza vaccine is recommended for all children undergoing treatment for cancer 11. However, up to one‐third of pediatric oncologists do not recommend yearly influenza vaccination 12, 13, with poor uptake (27–55%) identified in children with cancer 14, 15, 16, 17. The lack of knowledge regarding benefit of the influenza vaccine in this population has been identified as one of the reasons for poor compliance 14, 17. The absence of literature correlating clinical outcome with immune response following influenza vaccination in children with cancer may reflect this lack of knowledge 18. Given such findings, we undertook a prospective study to evaluate the immunogenicity and clinical effectiveness of the seasonal trivalent inactivated influenza vaccine in immunocompromised children receiving therapy for cancer, with the aim of providing evidence for annual influenza vaccination in this population and identifying risk factors that predict response.

## Methods

### Patient selection

Children between the ages of 6 months and 18 years who were receiving, or within 4 weeks from completion of immunosuppressive therapy for cancer were eligible. Recruitment was undertaken during the active influenza seasons of 2010 and 2011 (March–September) from the Department of Clinical Haematology and Oncology, Princess Margaret Hospital for Children (PMH) in Perth. PMH is the sole treatment center for all children with cancer in the state of Western Australia, which has a population of 2.6 million. Exclusion criteria included anaphylaxis to previous doses of any influenza vaccine, a history of egg anaphylaxis, receipt of intravenous immunoglobulin within the last 3 months, a neutrophil count of ≤0.5 × 10^9^/L, a history of Guillain–Barré syndrome and children having undergone autologous stem cell rescue or allogeneic hematopoietic stem cell transplant. Informed consent was obtained from the parents of each child prior to recruitment.

### Study design

Patients were vaccinated according to national Australian standards 19. Children <10 years of age, receiving influenza vaccine for the first time, were given two doses of the trivalent inactivated influenza vaccine 1 month apart. Children <10 years of age who had previously received influenza vaccine and children who were ten or older, were given a single dose of the vaccine. A 0.25 mL dose was administered to children <3 years of age, whereas those three or older were given 0.5 mL. The strains included in the vaccine for the 2010 and 2011 seasons were A/Perth/16/2009 (H3N2), A/California/7/2009 (H1N1), and B/Brisbane/60/2008 (B). Children were observed for 20 min after each vaccination for immediate vaccine‐related adverse events. Parents were given a daily diary for documentation of vaccine‐related adverse events occurring in the 7 days following vaccination and this information was collected from the parents at each visit.

Blood was taken prior to each vaccination and 4 weeks following the final vaccination to assess influenza‐specific immune responses. Following collection, blood samples were centrifuged and sera stored at −20°C. At the end of each season, the samples were sent to the World Health Organization (WHO) Collaborating Centre for Reference and Research on Influenza, Victorian Infectious Diseases Reference Laboratory (VIDRL) where influenza‐specific hemagglutinin inhibition (HI) antibody titers were performed against each vaccine strain by standardized assay 20.

Susceptibility to a vaccine‐like strain was defined as a prevaccination HI titer of <40. Seroprotection in an individual was defined as a postvaccination HI titer of ≥40. Seroconversion was defined as either a fourfold increase in HI antibody titer if the prevaccination titer was ≥10 or a rise in HI titer from <10 to ≥40 following vaccination 21. The percentage (95% confidence interval [CI]) of patients who individually met these criteria for seroprotection and seroconversion to each strain of the vaccine was calculated.

Criteria as established by the Committee for Proprietary Medicinal Products (CPMP) 22, were used to determine whether the vaccine was considered to elicit an effective overall immunogenic response in our immunocompromised population. According to these criteria, the influenza vaccine is considered effective if it meets one of the following three criteria: seroprotection in >70% of patients; seroconversion in >40% of patients; or a geometric mean fold increase (GMFI) >2.5. These criteria were used to calculate one‐sided *P* values in relation to the null hypotheses for overall seroprotection and seroconversion to each strain of the vaccine. GMFI was calculated for each strain as the geometric mean of the fold increase in antibody level after vaccination, with 95% CI and one‐sided P values estimated using a lognormal approximation for the distribution of antibody levels pre and postvaccination and the CPMP defined threshold of GMFI >2.5.

Multivariate logistic regression models were used to assess the influence of clinically relevant variables to predict seroconversion to each strain and complete seroconversion to all three strains. The variables analyzed within the model were sex (male, female), age groups as determined by the stratification according to the vaccination schedule (<3 years, 3–<10 years, 10–<18 years), tumor type (solid, hematological), treatment intensity in the 4 weeks preceding vaccination (low, high; classified according to anticipated extent and duration of immunosuppression (see Table S1) and lymphocyte count for age (normal range, less than lower limit of normal). Lower normal limits for absolute lymphocyte counts according to age were defined as 1.7 × 10^9^/L for children <5 years of age, 1.1 × 10^9^/L for 5–<10 years, and 1.0 × 10^9^/L for ≥10 years 23. The analyses were repeated on the subgroup of patients <10 years of age with the addition of vaccine doses received (one, two) as a variable in the model.

The standard procedure for all children with cancer in Western Australia who develop influenza‐like illness, is to present for a clinical review and a nasopharyngeal aspirate is taken. Influenza‐like illness is defined as an elevated temperature (≥37.5°C) or a clear history of fever (e.g., chills, rigors); the presence of at least one constitutional symptom from irritability, myalgia, headache, vomiting, diarrhea, or malaise; and the presence of at least one respiratory symptom from cough, sore throat, or rhinorrhea; with onset of symptoms occurring greater than 72 h after vaccine administration. The nasopharyngeal aspirates of all enrolled patients that were polymerase chain reaction positive for influenza were sent to the WHO Collaborating Centre for Reference and Research on Influenza, VIDRL, for culture and specific strain typing. Clinical effectiveness was assessed by comparing the proportion of laboratory confirmed influenza infection of vaccinated children on study with unvaccinated immunocompromised children receiving treatment for cancer who did not partake in the study at the time it was undertaken. Relative risk of infection comparing vaccinated and unvaccinated groups was estimated using log binomial regression, adjusting for age group and tumor type, with vaccine effectiveness calculated as 100 × (1 – relative risk). Clinical features of all children with cancer and laboratory proven influenza infection were documented and a qualitative assessment of clinical severity between vaccinated and unvaccinated patients was performed.

This study was approved by the Child and Adolescent Health Service Ethics Committee (Ethics Approval Number 1672/EP) with delegated authority from the PMH Institutional Review Board, within which the work was undertaken. It conforms to the provisions of the Declaration of Helsinki and the National Statement on Ethical Conduct in Human Research, Australian National Health and Medical Research Council. Statistical analyses were performed using SPSS Version 22.0: Armonk, NY, USA.

## Results

There were 100 patients enrolled in the study, of which 80% were susceptible to H3N2, 67% to H1N1, and 88% to B strain prior to the first vaccination. Patient characteristics are listed in Table 1. Seroprotection was achieved in 55% for H3N2, 61% for H1N1, and 41% for B strain following vaccination. Seroconversion occurred in 43% for H3N2, 43% for H1N1, and 33% for B strain. A significant response to the H3N2 (GMFI 4.56, 95% CI 3.19–6.52, *P *<* *0.01) and H1N1 (GMFI 4.44, 95% CI 3.19–6.19, *P *<* *0.01) strains was observed using CPMP criteria. Table 2 shows the immunological response to each strain.

**Table 1:** Patient demographics

Characteristic	Number of patients (*n* = 100)
Sex	
Male	63
Female	37
Age	
6 months to <3 years	15
3 to <10 years	53
10 to <18 years	32
Cancer type	
Hematological	63
Pre‐B acute lymphoblastic leukemia	39
T‐cell acute lymphoblastic leukemia	8
Acute myeloid leukemia	6
Non‐Hodgkin lymphoma	6
Hodgkin lymphoma	3
Langerhans cell histiocytosis	1
Solid	37
Central nervous system tumor	15
Wilms tumor	7
Ewing sarcoma	5
Rhabdomyosarcoma	4
Retinoblastoma	2
Germ cell tumor	2
Sex cord stromal tumor	1
Nasopharyngeal carcinoma	1
Dosing schedule	
One dose	67
Two doses	33

**Table 2:** Overall immunogenicity to trivalent inactivated influenza vaccine in immunocompromised children receiving treatment for cancer

Vaccine strain	GMFI (95% CI)	*P* value	Seroprotection % (95% CI)	*P* value	Seroconversion % (95% CI)	*P* value
H3N2 (A)	4.56 (3.19–6.52)	<0.01	55 (45.2–64.8)	>0.99	43 (33.3–52.7)	0.27
H1N1 (A)	4.44 (3.19–6.19)	<0.01	61 (51.4–70.6)	0.98	43 (33.3–52.7)	0.27
B	3.07 (2.17–4.36)	0.12	41 (31.4–50.6)	>0.99	33 (23.8–42.2)	0.92

GMFI, geometric mean fold increase.

The multivariate analysis of predictive variables revealed that children with solid tumors were significantly more likely to serorespond to each vaccine strain (H3N2: OR 7.39, 95% CI 2.42–22.53, *P *<* *0.01; H1N1: OR 2.90, 95% CI 1.02–8.23, *P* = 0.045; B: OR 3.75, 95% CI 1.25–11.24, *P* = 0.02) and to undergo complete seroconversion to all three strains (OR 6.03, 95% CI 1.56–23.29, *P *<* *0.01) compared to patients with hematological malignancies. Children with a lymphocyte count in the normal range for age were significantly more likely to serorespond to B strain (OR 2.97, 95% CI 1.07–8.28, *P* = 0.04) than those with a low lymphocyte count at vaccination. Children who received low intensity therapy in the 4 weeks preceding vaccination were significantly more likely to serorespond to the B (OR 3.16, 95% CI 1.09–9.18, *P* = 0.03) and H3N2 (OR 2.81, 95% CI 1.00–7.89, *P* = 0.049) strains compared with those who received high intensity therapy, with a trend for seroconversion demonstrated for H1N1 (OR 2.36, 95% CI 0.91–6.09, *P* = 0.08). Table 3 shows the multivariate analysis of factors predicting seroconversion. Multivariate analysis for the subgroup of patients <10 years of age revealed that vaccine naïve children who received two doses of the vaccine were significantly more likely to serorespond to each strain (H3N2: OR 6.08, 95% CI 1.56–23.63, *P *<* *0.01; H1N1: OR 6.03, 95% CI 1.74–20.90, *P *<* *0.01; B: OR 14.72, 95% CI 2.80–77.36, *P *<* *0.01), and to serorespond to all three strains (OR 14.71, 95% CI 1.27–170.2, *P* = 0.03), than children who had been vaccinated in previous seasons and only received one dose of the vaccine.

**Table 3:** Multivariate analysis of factors predicting seroconversion to trivalent inactivated influenza vaccine in immunocompromised children receiving treatment for cancer

Variable	H3N2 (A)		H1N1 (A)		B	
	OR (95% CI)	*P* value	OR (95% CI)	*P* value	OR (95% CI)	*P* value
Sex						
Female	1		1		1	
Male	0.80 (0.29–2.19)	0.66	2.30 (0.83–6.37)	0.11	0.65 (0.24–1.78)	0.40
Age						
6 months to <3 years	1		1		1	
3 to <10 years	0.92 (0.19–4.42)	0.91	0.36 (0.07–1.73)	0.20	2.41 (0.50–11.59)	0.27
10 to <18 years	2.27 (0.45–11.48)	0.32	0.34 (0.07–1.70)	0.19	4.24 (0.87–20.69)	0.07
Tumor type						
Hematological	1		1		1	
Solid	7.39 (2.42–22.53)	<0.01	2.90 (1.02–8.23)	0.045	3.75 (1.25–11.24)	0.02
Treatment intensity						
High	1		1		1	
Low	2.81 (1.00–7.89)	0.049	2.36 (0.91–6.09)	0.08	3.16 (1.09–9.18)	0.03
Lymphocyte count						
Low	1		1		1	
Normal range	1.66 (0.59–4.66)	0.34	1.93 (0.72–5.16)	0.19	2.97 (1.07–8.28)	0.04

The incidence of laboratory proven influenza infection in the vaccinated study population was 2% (*n* = 2/100), whereas in the unvaccinated control population there was an incidence of 6.8% (*n* = 11/161), giving an adjusted estimated vaccine effectiveness of 72% (95% CI −26– 94%). Of the children with laboratory proven influenza, there were 10 with acute lymphoblastic leukemia (ALL), including both vaccinated study patients, and individual patients with acute myeloid leukemia, Langerhans cell histiocytosis, and osteosarcoma. The two patients in the study had laboratory confirmed influenza B infection. Both patients failed to mount an immunological response to this strain with postvaccination HI titers <40. Unvaccinated children with influenza had an increased length of hospital admission (5.1 vs. 4 days, mean) and delay in the delivery of scheduled chemotherapy (4.5 vs. 0.5 days, mean) compared to vaccinated children with influenza. One unvaccinated control required supplemental oxygen for 3 days, however, there were no severe complications of influenza illness, such as admission to intensive care or death.

There were no vaccine‐related serious adverse events. Reactogenicity, that was considered attributable to the vaccine, occurred in four children, who all developed fever within 24 h of receiving the vaccine, with no other cause identified. All four children required brief inpatient admissions for empiric antibiotic therapy until the fever subsided. The parent reported reactogenicity rate was 3% (*n* = 4/133 vaccinations).

## Discussion

Influenza is a common respiratory pathogen associated with significant morbidity and mortality in children undergoing therapy for cancer 5, 6, 7, 8, 9, 10, yet recommendation for and uptake of the seasonal influenza vaccine remains suboptimal 12, 13, 14, 15, 16, 17. Lack of knowledge regarding the benefit of the influenza vaccine in this population has been cited as a reason for poor uptake 14, 17. Our study provides evidence that the trivalent inactivated influenza vaccine is safe, immunogenic and provides clinical protection for immunosuppressed children receiving treatment for cancer.

Our study confirms that immunocompromised children receiving treatment for cancer are able to mount a clinically significant immune response to the trivalent inactivated influenza vaccine. A wide range for seroprotection and seroconversion following administration of the trivalent influenza vaccine has been reported in this setting (summarized in Table 4) 24, 25, 26, 27, 28, 29, 30, 31, 32, 33, 34, 35. In our cohort, immunogenicity was toward the middle of these ranges: seroprotection H3N2 55% (range from published literature: 25–92%), H1N1 61% (5–96%), and B 41% (15–87%); seroconversion H3N2 43% (25–78%), H1N1 43% (16–84%), and B 33% (12–60%). Although this is reassuring, comparison between studies should be interpreted with caution due to the differences in methodology, season, and vaccine composition. In particular, different vaccination schedules, differing definitions of seroprotection and seroconversion, inclusion restricted to variables such as tumor type, and a broad range of methods to conduct statistical analyses have been used. The studies have been conducted over a large chronological time span, thus variations in susceptibility, differing circulating influenza strains, and vaccine composition need to be considered. Our study is only the second performed in the Southern Hemisphere using Southern Hemisphere influenza vaccine formulation, with the other study undertaken more than 30 years ago 35.

**Table 4:** Summary of the published literature assessing immunogenicity and variables predicting seroconversion to the trivalent inactivated influenza vaccine in immunocompromised children receiving treatment for cancer

Study (Publication year)	No.	Cancer type	Hemisphere	Influenza season	Seroprotection (%)			Seroconversion (%)			Variables predicting superior seroconversion
					H3N2	H1N1	B	H3N2	H1N1	B	
Kotecha (Current study)	100	All types	Southern	2010–2011	55	61	41	43	43	33	Solid > Hematological tumors
											Children <10 years of age: 2 > 1 dose schedule
											B and H3N2: Low > High intensity therapy in 4 weeks prior to vaccination
											B: Lymphocyte count in normal range for age at vaccination
Ottoffy (2014) 24	27	All types	Northern	2009–2011	78	48	15	37	22	22	Lymphocyte count >1.0 × 10^9^/L at vaccination
											Normal IgG at vaccination
Kersun (2013) 25	177	All types	Northern	2006–2010	_	_	_	_	_	_	H1N1: Solid tumors > ALL
											Induction phase of therapy in ALL
											Higher CD4 and CD8 influenza‐specific T‐cell responses in ALL
											B: Higher baseline B‐cell count in ALL
Carr (2011) 26	26	All types	Northern	2008–2009	92	73	31	46	27	12	No significant variables identified
Shahgholi (2010) 27	32	ALL Maintenance	Northern	2007–2008	63	43	26	41	56	59	Not assessed
Reilly (2010) 28	89	All types	Northern	2006–2008	_	_	_	34	22	35	Predictive variables assessed as part of Kersun et al. (2013) 25
Bektas (2007) 29	45	Solid^a^	Northern	2003–2004	98	96	87	78	84	60	B: Within 6 months from completion of therapy > On treatment
Matsuzaki (2005) 30	44^b^	All types	Northern	2003‐2004	25	42	29	25	38	33	H1N1 and H3N2: Completion of chemotherapy > On treatment
											H1N1 and B: IgG >690 mg/dL at vaccination
											B: White blood cell count >5 × 10^9^/L at vaccination
Chisholm (2005) 31	65	Solid^a^	Northern	2001‐2003	77	60	48	33	52	51	H1N1: Lymphocyte count >1.0 × 10^9^/L at vaccination
Porter (2004) 32	20	ALL Maintenance	Northern	2001‐2002	_	_	_	65	65	60	H1N1: 2 > 1 dose schedule
Hsieh (2002) 33	25	ALL Maintenance	Northern	2000‐2001	88	68	72	60	24	44	Not assessed
Chisholm (2001) 34	42	ALL^b^	Northern	1995‐1997	83	64	76	_	_	_	H1N1 and H3N2: Higher median age at vaccination
Feery (1979) 35, b	19	ALL Maintenance	Southern	1978	58	5	21	42	16	_	Not assessed

Includes lymphoma.

a Seroprotection and seroconversion given for up to 18 patients receiving chemotherapy; analysis of predictive variables undertaken on 44 patients of which 26 were up to 60 months following the completion of therapy.

Includes one patient with acute myeloid leukemia and three with solid tumors.

b Results shown for patients who received trivalent inactivated influenza vaccine containing H1N1, H3N2, and B strains. ALL, acute lymphoblastic leukemia.

The CPMP have defined criteria to assess whether influenza vaccines are effective within a population. The vaccine can be considered immunologically effective in our cohort as it satisfied criteria for GMFI. Although the vaccine did not meet the numerical threshold for seroprotection and statistical significance was not achieved for seroconversion, this should be taken in the context of how the criteria have been defined and not interpreted as vaccine failure. Although the CPMP criteria have been used to assess influenza vaccines in children, they are more specific to adults. In addition, the CPMP criteria have not been defined according to an immunocompromised population. This highlights the need for revised definitions, which take into account age and immune competence, to determine whether influenza vaccines are effective within specific populations.

Our study is the first to identify that children with solid tumors mount a significantly higher immune response to each strain individually and all three strains collectively compared to those with hematological malignancies. A previous study identified superior seroconversion to the H1N1 strain in children with solid tumors compared to those with ALL 25, and the study reporting the highest seroprotection and seroconversion rates in children with cancer was undertaken in children with solid tumors 29 (Table 4). This difference can be explained by the direct adverse effect of hematological malignancies on the immune system, as well as therapy directed toward continuous myelosuppression for leukemia, whereas treatment for solid tumors is generally shorter and cyclical in nature. The influence of treatment on effector cells of the immune system has been examined previously, demonstrating prolonged suppression on the B‐cell compartment in children receiving therapy for ALL compared with solid tumors 25.

Additional factors identified as predicting seroconversion from prior studies include higher white cell count, lymphocyte count, or IgG levels; increasing age; induction phase of therapy in ALL; and following completion of therapy (Table 4) 24, 25, 29, 30, 31, 34, which can all be considered correlates of underlying immune function. This is further emphasized by our results identifying a significantly superior seroconversion toward the B strain in children with a lymphocyte count in the normal range for age at vaccination and toward the B and H3N2 strains in children who received low compared with high intensity chemotherapy in the 4 weeks preceding vaccination. These correlates can be used as a guide to tailor the timing of vaccination on an individual basis, with optimal timing occurring prior to high intensity therapy, with normal lymphocyte counts and IgG levels in the patient. However, waiting for optimal conditions in an individual patient should not be at the expense of timely vaccination, given the risk of influenza to an unvaccinated immunocompromised child, the low chance of satisfying all predictive criteria at any one time in this population and that the vaccine remains safe and effective in children undergoing therapy for cancer despite immunosuppression.

Multivariate analysis of the subgroup of children <10 years of age has shown that vaccine naïve patients who received two doses of the vaccine were significantly more likely to seroconvert to each strain individually and all three strains collectively, than those vaccinated in previous seasons receiving one dose. This finding has previously been limited to seroconversion to the H1N1 strain in a small group of children with ALL 32. Our findings provide conclusive evidence to recommend that all immunocompromised children <10 years of age undergoing therapy for cancer should receive two age‐appropriate doses of the vaccine, regardless of prior vaccination history.

We have demonstrated that the trivalent inactivated influenza vaccine is clinically effective in immunocompromised children receiving treatment for cancer, with an adjusted estimated vaccine effectiveness of 72% (95% CI −26–94%). During the same influenza seasons, the estimated vaccine effectiveness in healthy children <5 years of age, recruited onto the Western Australian Influenza Vaccine Effectiveness study at PMH 36, was 60% (95% CI−70–91%); and for children <18 years of age presenting to general practitioners in Western Australia, as part of the Western Australian sentinel medical practice surveillance system for influenza 37, was 82% (95% CI 17–96%). The clinical effectiveness of the trivalent inactivated influenza vaccine in immunocompromised children receiving therapy for cancer was therefore comparable to geographically matched children during the same influenza seasons.

The average length of hospital admission and delay in the delivery of scheduled chemotherapy was noted to be greater in unvaccinated compared with vaccinated children with laboratory proven influenza infection. Although this study was not adequately powered for clinical features of influenza infection as an endpoint, these observations concur with outcomes of previous studies 6, 7, 38. There were no serious adverse events and a parent reported reactogenicity rate of 3%, which is comparable to the low rates in the literature 24, 27, 29, 30, 31, further supporting the safety of the trivalent inactivated influenza vaccine in immunocompromised children receiving therapy for cancer.

There are several limitations upon which this study is based. Despite recruiting a large number of children in comparison to contemporary published studies, the statistical analyses were limited by patient number and may explain the lack of significance for some of the analyses. The relative rarity of childhood cancer and the low incidence of laboratory proven influenza contribute to this limitation. Future studies should focus on recruiting larger numbers of patients, which may be facilitated through the conduct of large collaborative multicenter trials. To provide additional strength to the data, future studies should also consider prospective comparison of immunogenicity and clinical effectiveness to healthy age‐matched controls. It has been shown that children on maintenance therapy for ALL have an inferior but acceptable immune response when compared to healthy controls 27, 32, however, these findings have not been extended to other tumor types or other phases of ALL therapy. Finally, there is potential for variable exposure to influenza between the study and control population. This remains one of the generic limitations of testing clinical effectiveness in all influenza studies. It is also possible for test practices to vary between study and control populations, however, we do not expect this to influence the results of our study as children receiving therapy for cancer on our unit all routinely present for an assessment and nasopharyngeal aspirate if they develop influenza‐like illness.

In conclusion, our data demonstrate that the trivalent inactivated influenza vaccine is safe, immunogenic, and provides clinical protection in immunosuppressed children receiving therapy for cancer. On the basis of these outcomes, we recommend administration of the inactivated influenza vaccination on an annual basis for children undergoing treatment for cancer. An age‐appropriate two‐dose schedule should be administered to all children <10 years of age, regardless of prior vaccination history. The optimal time for immunization is prior to high intensity therapy, with response more likely in individuals with normal lymphocyte counts and IgG levels. These clinical correlates are reflective of underlying immune function and can be used as a guide to tailor the timing of vaccination on an individual basis, although should not result in vaccination delays. Larger studies are required to further validate the variables predicting seroconversion and to determine the benefit of a two‐dose schedule in all immunocompromised children receiving therapy for cancer regardless of age and prior vaccination history.

## Conflict of Interest

None declared.

## Supporting information


**Table S1.** Classification of treatment according to intensity.

## References

[1] Smith, M. A. , S. F. Altekruse , P. C. Adamson , G. H. Reaman , and N. L. Seibel . 2014 Declining childhood and adolescent cancer mortality. Cancer 120:2497–2506. 24853691 10.1002/cncr.28748 PMC4136455

[2] Hunger, S. P. , X. Lu , M. Devidas , B. M. Camitta , P. S. Gaynon , N. J. Winick , et al. 2012 Improved survival for children and adolescents with acute lymphoblastic leukemia between 1990 and 2005: a report from the children's oncology group. J. Clin. Oncol. 30:1663–1669. 22412151 10.1200/JCO.2011.37.8018 PMC3383113

[3] Youlden, D. R. , P. D. Baade , P. C. Valery , L. J. Ward , A. C. Green , and J. F. Aitken . 2012 Childhood cancer mortality in Australia. Cancer Epidemiol. 36:476–480. 22739323 10.1016/j.canep.2012.06.001

[4] Bosetti, C. , P. Bertuccio , L. Chatenoud , E. Negri , F. Levi , and C. La Vecchia . 2010 Childhood cancer mortality in Europe, 1970–2007. Eur. J. Cancer 46:384–394. 19818600 10.1016/j.ejca.2009.09.011

[5] Cooksley, C. D. , E. B. Avritscher , B. N. Bekele , K. V. Rolston , J. M. Geraci , and L. S. Elting . 2005 Epidemiology and outcomes of serious influenza‐related infections in the cancer population. Cancer 104:618–628. 15973737 10.1002/cncr.21203

[6] Feldman, S. , R. G. Webster , and M. Sugg . 1977 Influenza in children and young adults with cancer: 20 cases. Cancer 39:350–353. 576210 10.1002/1097-0142(197701)39:1<350::aid-cncr2820390153>3.0.co;2-8

[7] Tasian, S. K. , J. R. Park , E. T. Martin , and J. A. Englund . 2008 Influenza‐associated morbidity in children with cancer. Pediatr. Blood Cancer 50:983–987. 18240170 10.1002/pbc.21472 PMC4511372

[8] Kersun, L. S. , S. E. Coffin , K. H. Leckerman , M. Ingram , and A. F. Reilly . 2010 Community acquired influenza requiring hospitalization: vaccine status is unrelated to morbidity in children with cancer. Pediatr. Blood Cancer 54:79–82. 19743304 10.1002/pbc.22228

[9] Carr, S. B. , E. E. Adderson , H. Hakim , X. Xiong , X. Yan , and M. Caniza . 2012 Clinical and demographic characteristics of seasonal influenza in pediatric patients with cancer. Pediatr. Infect. Dis. J. 31:e202–e207. 22772169 10.1097/INF.0b013e318267f7d9 PMC3473140

[10] Esposito, S. , V. Cecinati , B. Scicchitano , G. C. Delvecchio , N. Santoro , D. Amato , et al. 2010 Impact of influenza‐like illness and effectiveness of influenza vaccination in oncohematological children who have completed cancer therapy. Vaccine 28:1558–1565. 20003924 10.1016/j.vaccine.2009.11.055 PMC7172348

[11] Committee on Infectious Diseases . 2014 Recommendations for prevention and control of influenza in children, 2014–2015. Pediatrics 134:e1503–e1519. 25246619 10.1542/peds.2014-2413

[12] Porter, C. C. , K. A. Poehling , R. Hamilton , H. Frangoul , and W. O. Cooper . 2003 Influenza immunization practices among pediatric oncologists. J. Pediatr. Hematol. Oncol. 25:134–138. 12571465 10.1097/00043426-200302000-00010

[13] Crawford, N. W. , J. A. Heath , and J. P. Buttery . 2007 Immunisation practices of paediatric oncologists: an Australasian survey. J. Paediatr. Child Health 43:593–596. 17608646 10.1111/j.1440-1754.2007.01162.x

[14] Hale, K. , and D. Isaacs . 2006 Survey of influenza immunisation uptake in ‘high risk’ children. J. Paediatr. Child Health 42:321. 16749195 10.1111/j.1440-1754.2006.00865.x

[15] Rees, H. , M. Andrews , S. Broster , J. Nicholson , R. Skinner , and J. Chisholm ; Supportive Care Group of the Children's Cancer Leukaemia Group . 2010 Influenza vaccination during cancer therapy. Arch. Dis. Child. 95:569–570. 20551204 10.1136/adc.2009.169078

[16] Crawford, N. W. , J. A. Heath , D. Ashley , P. Downie , and J. P. Buttery . 2010 Survivors of childhood cancer: an Australian audit of vaccination status after treatment. Pediatr. Blood Cancer 54:128–133. 19785022 10.1002/pbc.22256

[17] Esposito, S. , P. Marchisio , R. Droghetti , L. Lambertini , N. Faelli , S. Bosis , et al. 2006 Influenza vaccination coverage among children with high‐risk medical conditions. Vaccine 24:5251–5255. 16621177 10.1016/j.vaccine.2006.03.059

[18] Goossen, G. M. , L. C. Kremer , and M. D. van de Wetering . 2013 Influenza vaccination in children being treated with chemotherapy for cancer. Cochrane Database Syst. Rev. 8:CD006484. 23904194 10.1002/14651858.CD006484.pub3 PMC6466690

[19] Australian Technical Advisory Group on Immunisation . 2008 The Australian Immunisation Handbook, 9th ed Australian Government Department of Health, Canberra.

[20] WHO Global Influenza Surveillance Network . 2011 Manual for the laboratory diagnosis and virological surveillance of influenza. Available at http://whqlibdoc.who.int/publications/2011/9789241548090_eng.pdf?ua=1 (accessed 15 September 2015).

[21] Fiore, A. E. , C. B. Bridges , J. M. Katz , and N. J. Cox . 2013 Inactivated influenza vaccines Pp. 257–293 *in* Plotkin S., Orenstein W. and Offit P. A., eds. Vaccines, 6th ed. Elsevier, Philadelphia, PA.

[22] Committee for Proprietary Medicinal Products (CPMP) . Note for guidance on harmonisation of requirements for influenza vaccines. CPMP/BWP/214/96. European Agency for the Evaluation of Medicinal Products (EMEA), March 1997.

[23] Comans‐Bitter, W. M. , R. de Groot , R. van den Beemd , H. J. Neijens , W. C. Hop , K. Groeneveld , et al. 1997 Immunophenotyping of blood lymphocytes in childhood. Reference values for lymphocyte subpopulations. J. Pediatr. 130:388–393. 9063413 10.1016/s0022-3476(97)70200-2

[24] Ottoffy, G. , P. Horvath , L. Muth , A. Solyom , M. Garami , G. Kovacs , et al. 2014 Immunogenicity of a 2009 pandemic influenza virus A H1N1 vaccine, administered simultaneously with the seasonal influenza vaccine, in children receiving chemotherapy. Pediatr. Blood Cancer 61:1013–1016. 24395342 10.1002/pbc.24893

[25] Kersun, L. S. , A. Reilly , S. E. Coffin , J. Boyer , E. T. Luning Prak , K. McDonald , et al. 2013 A prospective study of chemotherapy immunologic effects and predictors of humoral influenza vaccine responses in a pediatric oncology cohort. Influenza Other Respir. Viruses 7:1158–1167. 23199016 10.1111/irv.12058 PMC4634289

[26] Carr, S. , K. J. Allison , L. A. Van De Velde , K. Zhang , E. Y. English , A. Iverson , et al. 2011 Safety and immunogenicity of live attenuated and inactivated influenza vaccines in children with cancer. J. Infect. Dis. 204:1475–1482. 21949042 10.1093/infdis/jir561

[27] Shahgholi, E. , M. A. Ehsani , P. Salamati , A. Maysamie , K. Sotoudeh , and T. Mokhtariazad . 2010 Immunogenicity of trivalent influenza vaccine in children with acute lymphoblastic leukemia during maintenance therapy. Pediatr. Blood Cancer 54:716–720. 20205258 10.1002/pbc.22421

[28] Reilly, A. , L. S. Kersun , K. McDonald , A. Weinberg , A. F. Jawad , and K. E. Sullivan . 2010 The efficacy of influenza vaccination in a pediatric oncology population. J. Pediatr. Hematol. Oncol. 32:e177–e181. 20485201 10.1097/MPH.0b013e3181d869f3

[29] Bektas, O. , C. Karadeniz , A. Oguz , S. Berberoglu , N. Yilmaz , and C. Citak . 2007 Assessment of the immune response to trivalent split influenza vaccine in children with solid tumors. Pediatr. Blood Cancer 49:914–917. 17262793 10.1002/pbc.21106

[30] Matsuzaki, A. , A. Suminoe , Y. Koga , N. Kinukawa , K. Kusuhara , and T. Hara . 2005 Immune response after influenza vaccination in children with cancer. Pediatr. Blood Cancer 45:831–837. 16007602 10.1002/pbc.20470

[31] Chisholm, J. , K. Howe , M. Taj , and M. Zambon . 2005 Influenza immunisation in children with solid tumours. Eur. J. Cancer 41:2280–2287. 16143516 10.1016/j.ejca.2005.07.006

[32] Porter, C. C. , K. M. Edwards , Y. Zhu , and H. Frangoul . 2004 Immune responses to influenza immunization in children receiving maintenance chemotherapy for acute lymphoblastic leukemia. Pediatr. Blood Cancer 42:36–40. 14752792 10.1002/pbc.10459

[33] Hsieh, Y. C. , M. Y. Lu , C. L. Kao , B. L. Chiang , D. T. Lin , K. S. Lin , et al. 2002 Response to influenza vaccine in children with leukemia undergoing chemotherapy. J. Formos. Med. Assoc. 101:700–704. 12517044

[34] Chisholm, J. C. , T. Devine , A. Charlett , C. R. Pinkerton , and M. Zambon . 2001 Response to influenza immunisation during treatment for cancer. Arch. Dis. Child. 84:496–500. 11369567 10.1136/adc.84.6.496 PMC1718812

[35] Feery, B. J. , R. N. Matthews , M. G. Evered , and H. A. Gallichio . 1979 Antibody responses to influenza virus vaccine in patients with acute lymphocytic leukaemia. Aust. Paediatr. J. 15:177–180. 293160 10.1111/j.1440-1754.1979.tb01220.x

[36] Blyth, C. C. , P. Jacoby , P. V. Effler , H. Kelly , D. W. Smith , C. Robins , et al. 2014 Effectiveness of trivalent flu vaccine in healthy young children. Pediatrics 133:e1218–e1225. 24753525 10.1542/peds.2013-3707

[37] Levy, A. , S. G. Sullivan , S. S. Tempone , K. L. Wong , A. K. Regan , G. K. Dowse , et al. 2014 Influenza vaccine effectiveness estimates for Western Australia during a period of vaccine and virus strain stability, 2010 to 2012. Vaccine 32:6312–6318. 25223268 10.1016/j.vaccine.2014.08.066

[38] Kempe, A. , C. B. Hall , N. E. MacDonald , H. R. Foye , K. A. Woodin , H. J. Cohen , et al. 1989 Influenza in children with cancer. J. Pediatr. 115:33–39. 2738793 10.1016/s0022-3476(89)80325-7

