# Supplementary material for: Immunogenicity and clinical effectiveness of the trivalent inactivated influenza vaccine in immunocompromised children undergoing treatment for cancer
Source: Cancer Med. 2015 Dec 29;5(2):285–93. doi: 10.1002/cam4.596 (PMC4735770; doi:10.1002/cam4.596)
Supplement: Supplementary file 1 — Table S1. Classification of treatment according to intensity. [file CAM4-5-285-s001.docx]

**Supplementary Table 1. Classification of treatment according to intensity**

| **Tumor type** | | **High intensity** | **Low intensity** |
| --- | --- | --- | --- |
| Acute Lymphoblastic Leukemia | | Induction  Augmented Consolidation  Delayed Intensification | Standard Consolidation  Interim Maintenance  Maintenance |
| Acute Myeloid Leukemia | | All therapy except Maintenance for APML | Maintenance for APML |
| Non Hodgkin Lymphoma | | Cytarabine/Methotrexate (CYM) | Doxorubicin/Mercaptopurine/Prednisone |
| Hodgkin Lymphoma | | Doxorubicin/Bleomycin/Vincristine/Etoposide – Prednisone/Cyclophosphamide (ABVE-PC) |  |
| Langerhans Cell Histiocytosis | |  | Vinblastine/Prednisone |
| Central Nervous System Tumors | Medulloblastoma | Lomustine/Cisplatin/Vincristine  Cyclophosphamide/Vincristine |  |
|  | Ependymoma | Vincristine/Carboplatin/Cyclophosphamide/Etoposide |  |
|  | High grade glioma | Cyclophosphamide/Carboplatin/Etoposide | Temozolomide |
|  | Low grade glioma |  | Vinblastine  Bevacizumab/Irinotecan  Carboplatin/Vincristine |
| Wilms Tumor | |  | Vincristine/Actinomycin D  Vincristine/Actinomycin D/Doxorubicin |
| Ewing Sarcoma | | Vincristine/Ifosfamide/Doxorubicin/Etoposide (VIDE)  Vincristine/Doxorubicin/Cyclophosphamide – Ifosfamide/Etoposide (VDC-IE) |  |
| Rhabdomyosarcoma | | Vincristine/Actinomycin D/Cyclophosphamide (VAC) | Vincristine/Irinotecan (VI) |
| Retinoblastoma | |  | Carboplatin/Etoposide/Vincristine (CEV) |
| Germ Cell/  Sex Cord Stromal Tumor | | Bleomycin/Etoposide/Cisplatin (BEP) |  |
| Nasopharyngeal Carcinoma | | Cisplatin/5-Fluorouracil |  |
